# Noninvasive intracranial pressure profile in 31 patients submitted to fullendoscopic spine surgery

**DOI:** 10.1590/acb396424

**Published:** 2024-09-20

**Authors:** André Tosta Ribeiro, Marcelo Campos Moraes Amato, Ricardo Santos de Oliveira

**Affiliations:** 1 Universidade de São Paulo – Faculdade de Medicina de Ribeirão Preto – Departmento de Cirurgia e Anatomia – Ribeirão Preto (SP) – Brazil.

**Keywords:** Intracranial Pressure, Spine, Endoscopy, Cerebrospinal Fluid

## Abstract

**Purpose:**

Full-endoscopic spine surgery (FESS) is associated with specific complications, possibly linked to increased intracranial pressure (ICP) resulting from continuous saline infusion into the epidural space. This study aimed to assess the impact of saline irrigation and its correlation with noninvasively obtained ICP waveform changes.

**Methods:**

Patients undergoing FESS between January 2019 and November 2020 were included. Noninvasive ICP (n-ICP) monitoring utilized an extracranial strain sensor generating ICP waveforms, from which parameters P2/P1 ratio and time to peak (TTP) values were derived and correlated to irrigation and vital parameters. Documentation occurred at specific surgical intervals (M0–preoperatively; M1 to M4–intraoperatively; M5–postoperatively). Mixed-model analysis of variance and multiple comparisons tests were applied, with M0 as the baseline.

**Results:**

Among 31 enrolled patients, three experienced headaches unrelated to increased ICP at M5. The P2/P1 ratio and TTP decreased during surgery (*p* < 0.001 and *p* < 0.004, respectively). Compared to baseline, P2/P1 ratio and vital parameters remained significantly lower at M5. No significant differences were observed for fluid parameters throughout surgery.

**Conclusions:**

This study demonstrated a decline in the n-ICP parameters after anesthetic induction despite the anticipated increase in ICP due to constant epidural irrigation. The n-ICP parameters behaved independently of fluid parameters, suggesting a potential protective effect of anesthesia.

## Introduction

Individuals with lower back or radicular pain resulting from lumbar spine disorders such as disc herniation or spinal canal stenosis are frequently seen in medical offices and emergency care units. Approximately 85 to 90% of these individuals are managed conservatively without major problems; however, 10 to 15% of them require surgical resolution^1^.

While lumbar microdiscectomy (MD) remains a stablished surgical treatment^2^^,^ 3, and full-endoscopic spine surgery (FESS) has gained greater acceptance among spine surgeons. This can be attributed to ongoing surgical technique improvement and diffusion of specialized training centers. In addition, when compared to MD, FESS has better results concerning blood loss, length of hospital stay, adjacent tissue injuries and infection^2^^–^ 5.

Despite the evident advantages of FESS, some specific complications, such as transient or persistent headache, neck pain, hearing loss, lower limb paresthesias^6^, visual impairment, retinal hemorrhage^7^^,^ 8, and even epileptic seizures^9^^,^ 10, have been reported in the literature. An increase in intracranial pressure (ICP) resulting from the continuous infusion of saline solution into the epidural space is suggested as a plausible explanation, as recently demonstrated by Amato et al.^11^ in an experimental study with pigs. However, the actual occurrence of intracranial hypertension during FESS in humans remains unproven, mostly due to technical challenges in obtaining reliable ICP data.

Considering the diversity of noninvasive indirect ICP methods described^12^, such as pressure on the anterior fontanelle, diameter of the optic nerve sheath, venous ophthalmodynamometry, tympanometry, tissue resonance analysis, tonometry, acoustoelasticity, otoacoustic emissions, transcranial Doppler, electroencephalogram, near-infrared spectroscopy and pupillometry, none, as of yet, is sufficiently accurate and effective for widespread use in clinical practice^13^. However, ICP waveform analysis obtained noninvasively has been recently demonstrated to be a reliable method to understand whether ICP variation indeed occurs, as shown in other studies^14^^–^ 16.

**Figure 1:**
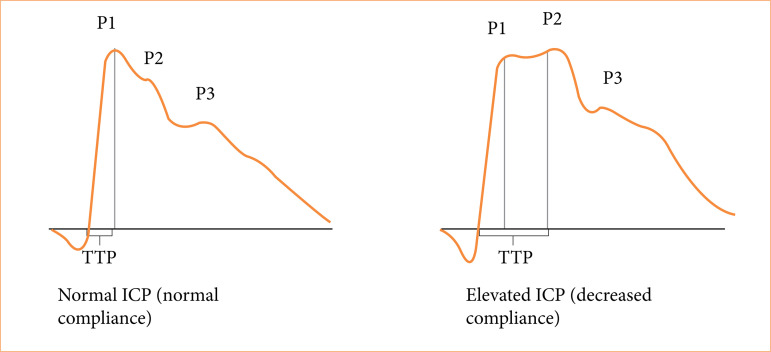
The intracranial pressure waveform.

The ICP morphology is usually divided into three primary peaks: the percussion wave (P1), the tidal wave (P2), and the dicrotic wave (P3)^17^^,^ 18. The time between the onset of the ICP wave and its highest value is named time to peak (TTP), considered as an useful value^19^. As known, an increase in the P2 component that exceeded P1 corresponded to an increase in the ICP^20^ and rounded shape of the ICP, with centering of the wave peak, could be related to intracranial hypertension (TTP value)^13^ (Fig. 1). In the last decade, many studies have supported the ICP waveform analysis for a myriad of diseases^13^^,^ 19 ^–^ 28 thus encouraging its use in evaluating ICP behavior in this study.

*The time-to-peak variable is the time length measured from the base of P1 until the highest peak, whether P1 or P2; P1: perfusion wave; P2: tidal wave; P3: dicroic wave; TTP: time to peak: ICP: intracranial pressure. Source: Elaborated by the authors.

Since continuous saline irrigation, as used in FESS, is known to cause ICP changes, the objective of the present study was to evaluate the P2/P1 ratio and the TTP parameters in the ICP waveform obtained noninvasively in individuals undergoing FESS.

## Methods

### Patients

A total of 31 individuals with indications for FESS were selected from January 2019 to November 2020 to participate in the study. The study was submitted for prior approval by the Research Ethics Committee of Hospital das Clínicas, School of Medicine of Ribeirão Preto, Universidade de São Paulo, under number 4.029.811. All patients were informed about the study by the researcher at the time of surgery and had their informed consent forms signed.

This is a prospective observational study.

The inclusion criteria were as follows: patients over 18 years old with lumbar disc herniation, lumbar spinal stenosis or lumbar foraminal stenosis. There was no restriction regarding cases of reoperation, nor any distinction made regarding the access route. The exclusion criteria were as follows: patients younger than 18 years old, the presence of any central nervous system disorder, previous cranial surgery and low-quality ICP waveform recording that prevented it from being read by the software.

All surgeries were performed using the percutaneous full-endoscopic technique, with sedation in cases of transforaminal access (using propofol, 3.5 mg/kg/h, or dexmedetomidine, 0.3 to 0.5 mcg/kg/min) and general anesthesia in cases of interlaminar access (using propofol, 3.5 mg/kg/h, or sevoflurane, 1.5% or 0.7 MAC, never combined endovenous and inhalatory sedatives). None of the individuals received any preanesthetic medication. All patients had a surgical risk classification of ASA 1 or 2.

Sedation was chosen for transforaminal access due to the risk of nerve root injury during disc access through Kambim’s triangle, a step performed exclusively under fluoroscopy^29^, wherein radicular pain should be avoided. For cases of interlaminar access, however, the risk of nerve damage was considered minimal, and general anesthesia was preferred.

During the surgical procedure, all subjects underwent continuous blood pressure, heart rate (HR) and ventilation monitoring, in addition to capnography and oximetry. Surgical site irrigation was performed using a Richard Wolf Fluid Control 2203 automatic pump. The ideal irrigation pressure (IP) and flow rate (FR) were determined by the surgeon at the time of surgery, and adjustments were made to obtain the best visual clarity for performing the surgery.

The individuals were placed in the ventral decubitus position with support cushions under the iliac crests and clavicles and a silicone support for the head to maintain head and spine alignment.

Vital parameter monitoring was initiated, followed by the administration of antibiotic prophylaxis. Finally, the noninvasive ICP (n-ICP) sensor was placed, and the signal quality was checked in real time on the monitor. Once the signal quality was visually good, sterile drapes were placed, and the surgery was started.

### Noninvasive intracranial pressure method

Noninvasive ICP monitoring was performed using an extracranial strain sensor (Brain4Care, Braincare Desenvolvimento e Inovação Tecnológica SA, São Carlos, SP, Brazil) secured to the scalp by a flexible acrylic strap firmly adjusted with a pressure ratchet. The sensor was placed on the right or left frontotemporal region at a point on the skull with optimal signal capture, approximately 5 cm above the tragus. There was no need for any preparation or hair removal prior to sensor placement.

**Figure 2:**
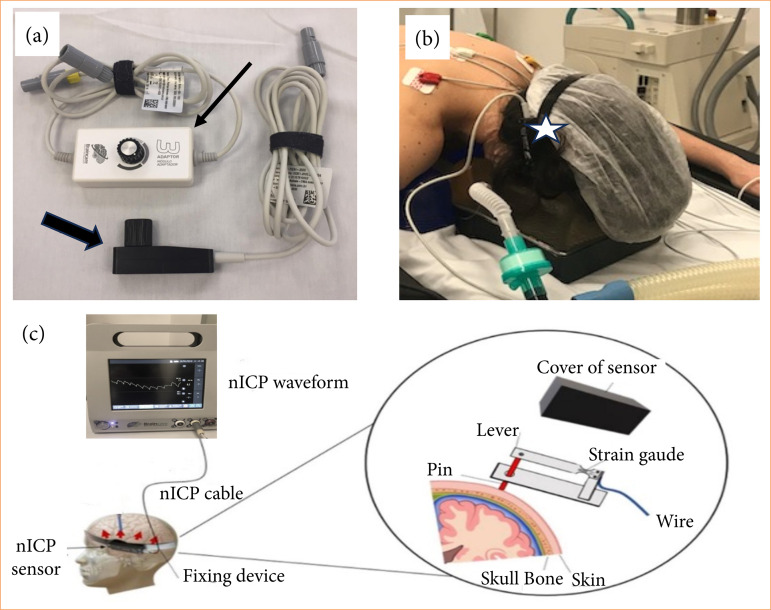
Devices used for noninvasive intracranial pressure (ICP) monitoring (BrainCare). **(a)** Thick black arrow: extensometer (sensor) used for measuring ICP, in contact with the individual’s scalp. Thin arrow: signal amplitude controller. **(b)** Star: acrylic band on the patient’s skull, with attached extensometer. **(c)** Infographic demonstrating the curve of intracranial pressure obtained in real-time during the surgical procedure, based on cranial pulsations detected by the sensor (enlarged figure).

This sensor detected skull deformations caused by changes in intracranial pressure^30^. It filtered, amplified, and digitalized the signal, obtained in millivolts (mV), passing through mathematical filtering with a cutoff frequency of 5 Hz, which enabled the generation of the appropriate graphic image, and then sent the data to a computer^31^. Such oscillations generate pulse waves correspondent to invasive ICP monitoring^30^^,^ 32 (Fig. 2).

nICP: noninvasive intracranial pressure. Source: Elaborated by the authors.

**Figure 3:**
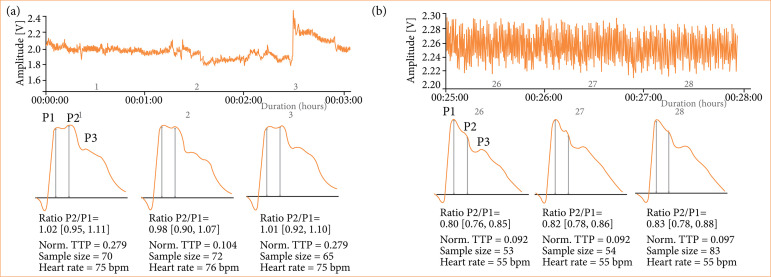
Graphics generated by Brain4Care Analytics in two distinct moments in the same individual (n = 17). **(a)** Report generated in the preoperative period. At the top, continuous record of cranial box pulsations depicted by the solid blue line, measured in Volts on the vertical axis. This continuous data is condensed into curves, representing 3-min intervals, as seen below. **(b)** A second report details the “closed channel” phase from the 26th to the 28th min, highlighting observable changes in the curve pattern. The P2/P1 ratio and TTP values are displayed at the bottom of each curve for both instances.

P1: perfusion wave; P2: tidal wave; P3: dicroic wave; TTP: time to peak: ICP: intracranial pressure. Source: Elaborated by the authors.

The ICP waveform was displayed by the hardware that came with the system. It was simultaneously encrypted on a memory card and then transferred to the online platform Brain4Care Analytics 1.0, which decrypted the signals and condensed each minute of recording into a single wave, from which the P2/P1 ratio and TTP values were extracted (Fig. 3). It is important to notice that, although the n-ICP waveform might appear visually satisfactory during real-time monitoring, data could be lost if the analytics program could not analyze the recorded waves.

### Monitoring and data analysis

**Figure 4:**
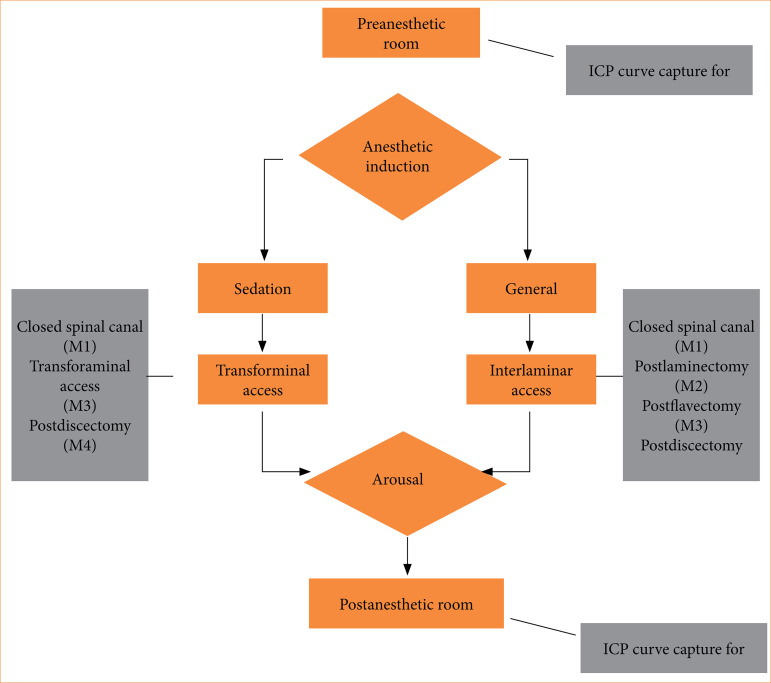
Flowchart illustrating the data collection time points, shown in the gray squares.

Source: Elaborated by the authors.

The data collection intervals followed the steps of the surgery, as illustrated in the flowchart in Fig. 4. There were three distinct moments to place the n-ICP sensor: in preoperative room, with patients lying down in the supine position and their reads inclined at 30 degrees (M0); during the surgery, after anesthesia and with patients in the prone position (M1, M2, M3, and M4); and finally in the postoperative room, with patients lying down in supine position and their reads inclined at 30 degrees. The signal quality of the n-ICP waveform had to be visually satisfactory before starting the recording.

The data were recorded and tabulated in a Microsoft Excel 2014 spreadsheet and analyzed using Minitab statistical software, version 18.1, with the significance level of *p* < 0.05.

Documentation occurred at each saline flow rate or pressure variation during the surgical procedure and at each specific surgical interval described. These data were compared with the preoperative values for each individual to determine the occurrence of loss of brain compliance. The irrigation time and the total amount of saline solution were also documented for subsequent correlation with the findings in cases of intracranial hypertension.

Longitudinal data were analyzed using a mixed-model analysis of variance (ANOVA), considering the *surgical interval* as a fixed factor with repeated measures and the *individual* as a random factor. For the analysis of the P2/P1 and TTP parameters, mixed-model ANOVA was also used to determine the impact of the *type of surgery, age*, and *surgical interval* parameters. When a factor was found to be statistically significant with repeated measures, the Bonferroni multiple comparison test was applied to determine which evaluations showed a significant difference compared to the baseline evaluation.

## Results

A total of 31 patients (16 males and 15 females) who underwent endoscopic spinal procedures were evaluated; 26 (83.9%) underwent interlaminar endoscopic surgery, and five (16.1%) underwent transforaminal endoscopic surgery (Table 1).

**Table 1:** The main study data is compiled for each selected individual.

N	Sex	Age	BMI	Access	Anesthesia	Mean FR (L/min)	Mean IP (mmHg)	Irrigation time (min)	Pain
1	M	37	25.1	TF	Sed	0.57	29.3	58	N
2	M	33	31	IL	Gen	0.5	85	101	N
3	M	56	25.3	IL	Gen	0.8	110	140	N
4	M	26	22.4	IL	Gen	0.6	95	100	N
5	F	63	26.3	IL	Gen	0.7	111.25	81	Y
6	F	35	29.3	IL	Gen	0.43	85	135	N
7	F	22	21	IL	Gen	0.68	102.5	143	N
8	M	58	35.3	TF	Sed	0.7	138.33	143	N
9	F	55	24.7	IL	Gen	0.68	83.13	90	Y
10	F	39	23.5	IL	Gen	0.88	101.88	83	N
11	F	53	24	IL	Gen	0.88	103.13	145	N
12	M	34	35.8	IL	Gen	0.87	102.5	172	N
13	M	93	25.4	IL	Gen	0.57	102.22	145	N
14	F	51	28.7	IL	Gen	0.7	82.5	112	N
15	F	27	20.1	IL	Gen	0.68	88.13	69	N
16	F	39	33.2	IL	Gen	0.68	87.5	101	N
17	F	31	25.9	IL	Gen	0.5	70	66	N
18	M	76	30.4	IL	Gen	0.67	82.5	182	N
19	M	28	27.2	IL	Gen	0.68	87.5	74	N
20	F	45	22.5	IL	Gen	0.7	93.75	71	N
21	M	28	25.6	IL	Gen	0.7	82.5	97	N
22	M	33	35.5	IL	Gen	0.7	68.33	58	N
23	F	32	19.8	IL	Gen	0.7	80.42	98	N
24	F	52	23	IL	Gen	0.58	74.38	151	N
25	F	44	25.4	TF	Sed	0.63	62.5	77	N
26	M	49	28	IL	Gen	0.7	70	94	N
27	M	46	25.8	IL	Gen	0.48	66.67	87	N
28	M	55	24.4	TF	Sed	1.2	88.33	49	N
29	F	42	35	IL	Gen	1.2	85	94	N
30	M	50	26.1	IL	Gen	1.2	87.5	110	N
31	M	47	26.9	TF	Sed	0.86	82.78	42	Y

N: patient number; BMI: body mass index; FR: flow rate; IP: irrigation pressure; M: male; F: female; IL: interlaminar; TF: transforaminal; Gen: general anesthesia; Sed: sedation; Y: yes; N: no. Source: Elaborated by the authors.

The individuals had a mean age of 44.5 ± 15.4 years old, ranging from 22 to 93. The mean body mass index (BMI) was 26.8 ± 4.5 kg/m^2^, ranging from 19.8 to 35.8 kg/m^2^. A total of 12 (38.7%) had some associated disease, and systemic arterial hypertension was the most frequent (eight of 12 individuals). None of them complained of chronic headaches.

The mean total infused saline solution volume was 13.3 ± 5.L (4 to 29 L) per surgery. In all cases, only 0.9% saline solution was used. The mean surgery time was 110 ± 40.97 min (55 to 240 min), and the mean irrigation time was 100 ± 35.8 min (42 to 182 min).

General anesthesia was performed with sevoflurane for two individuals; the other 24 received propofol. Among the patients who underwent sedation, four received dexmedetomidine hydrochloride, and one received propofol.

**Figure 5:**
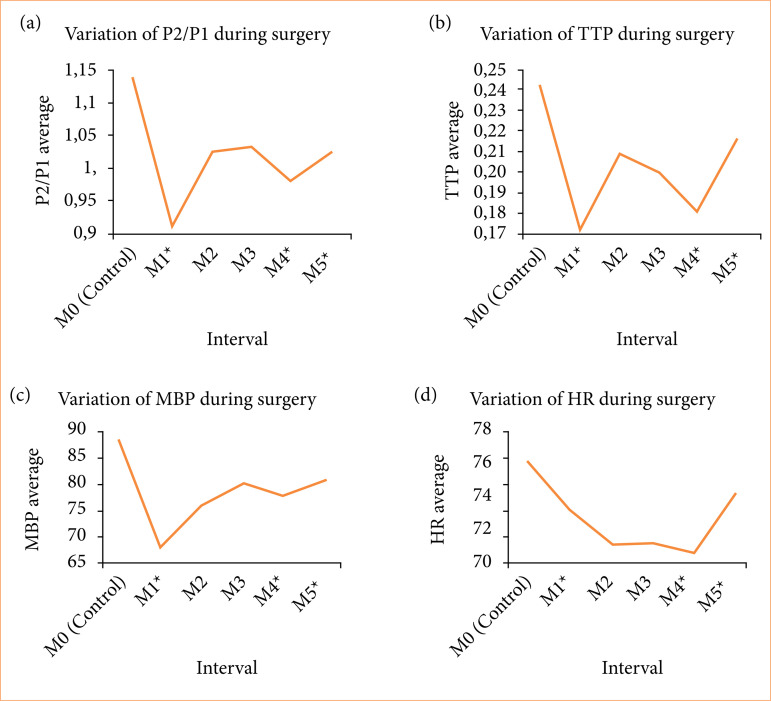
Graphics representing the variation of P2/P1, TTP, MBP and HR.

A linear correlation was found between P2/P1 ratio and age (in years), as an increase of 0.00525 in the P2/P1 ratio for each added year (*p* = 0.009). The P2/P1 ratio decreased according to the surgical interval (*p* < 0.001). The type of surgery performed (if interlaminar or transforaminal) did not significantly affect the P2/P1 ratio (*p* = 0.590) (Fig. 5a).

*Significant difference in relation to M0 (Bonferroni method); TTP: time to peak; MBP: mean blood pressure; HR: heart rate. Source: Elaborated by the authors.

The TTP, when related to the type of surgery (interlaminar or transforaminal) and age (in years), showed a decrease dependent only on the surgical interval (*p* = 0.004). The TTP did not vary significantly with age (*p* = 0.179) nor with the type of surgery (*p* = 0.954) (Fig. 5b).

There was a decrease in mean blood pressure (MBP) (*p* < 0.001) at all surgical intervals compared to baseline, whereas HR decreased from baseline at M2, M3 and M4 (Figs. 5c and 5d, respectively).

There were no differences in the documented 0.9% saline intraperitoneally (*p* = 0.103, with a mean pressure ranging from 84.37 to 90.19 mmHg) or FR (*p* = 0.071, with a mean pressure ranging from 0.7119 L/min to 0.7440 L/min) during the surgical procedures.

## Discussion

The epidemiological data of the present study regarding sex distribution, mean age, and mean BMI are consistent with the literature, which generally shows a higher incidence of lumbar disc herniation with increasing age and a higher frequency in males than in females^5^^,^ 33 ^–^ 35. The incidence of lumbar disc herniation also reflects the BMI distribution pattern in both the American population, in which more than 30% of individuals are obese^36^^,^ 37, and in the Brazilian population 25% are obese^38^.

The analysis of the P2/P1 ratio showed a mean value above 1.0 both preoperatively and postoperatively, what could be wrongly interpreted as chronic intracranial hypertension. However, other studies have shown a disproportionate increase in ICP considering a P2/P1 ratio above 0.8^14^^,^ 39 ^,^ 40. Fan et al.^40^ questioned the cutoff value of 0.8 because it was not related to the presence of intracranial hypertension symptoms.

In the present study, it was observed that the mean ratio remained consistently above 0.8, with no reported symptoms of intracranial hypertension. Within the concept of craniospinal compliance^41^, the spinal sub-compartment has been established as the one with the greatest capacity for volume accommodation^42^^,^ 43. As shown by Lee et al.^44^, abdominal compressions can reduce the cerebrospinal fluid volume of the spinal compartment via engorgement of the epidural venous plexus, a phenomenon that is even more pronounced in the presence of increased BMI^45^^–^ 47. This factor cannot be disregarded during spinal surgery, when the patients are kept in ventral decubitus, even if they are supported by cushions. However, patient positioning was done between M0 and M1, when a reduction at P2/P1 ratio was observed, instead of a higher expected value. A possible explanation for P2/P1 ratio above 1.0 at M0 could be a reduction in total compliance of the spinal sub-compartment, due to massive disc herniations or severe degenerative stenoses. Additionally, at M5, when the cerebrospinal fluid circulation in the spinal canal was reestablished, the P2/P1 ratio remained significantly lower than the baseline. The impact of patient age on pre-operative values of the P2/P1 ratio should be also considered. Since the mean age of patients was 44.5 years old, it is likely that the spinal sub-compartment played a significant role in craniospinal compliance^43^^,^ 46 ^,^ 48, particularly in conjunction with the reduction in spinal sub-compartment volume due to spinal disease. However, there were no clinical signs of ICP decompensation in any patient preoperatively.

More significant than the baseline, for both the P2/P1 and TTP values, there was an important decrease after anesthesia induction (M1) and again after discectomy (M4), and values persisted below baseline throughout the procedure. Together with the decrease in MBP and HR, the only plausible explanation is the effect of anesthesia. The absence of an invasive monitoring showing an absolute increase in ICP raises some doubts about the actual ICP effect of FESS. However, a decrease of P2/P1 and TTP, when an increase would be expected, gives us valuable information about the safety of the procedure and raises the hypothesis of a possible effect of anesthetic drugs to protect from intracranial hypertension during some direct epidural stimulus. Indeed, articles that report complications attributed to probable ICP increase have used local anesthesia and mild sedation or sevoflurane based general anesthesia^6^^–^ 10 ^,^ 49. Furthermore, propofol and opioids are known to be capable of reducing ICP and are preferred by most neuroanesthesiologists. The greater proportion of patients in this study were anesthetized with propofol or dexmedetomidine, both preferred for neurosurgical anesthesia, together with opioids, due to their vasoconstrictive properties^45^^,^ 50 ^–^ 52, as evidenced by reduced ICP and cerebral perfusion pressure^53^, and more effective in pain control^54^. Therefore, the use of anesthetic agents with direct action on brain autoregulation seems to be related to the ICP waveform behavior observed throughout the procedure.

The onset of symptoms reported in the literature, either intraoperatively or postoperatively, seems to be related to the volume of infused saline solution^5^, the time of exposure to irrigation^10^, and the type of anesthesia used^10^^,^ 55 ^,^ 56, all of which can overcome physiological ICP compensation mechanisms, such as the reduction of cerebral blood flow and cerebral perfusion pressure^10^^,^ 50 ^,^ 51 ^,^ 53. Again, the studies reporting headaches, neck pains, and severe neuro-ophthalmologic complications preferably used local and sevoflurane based anesthesia^6^^–^ 10 ^,^ 49. The small incidence of symptoms related to ICP rise in this series clinically supports the harmless variation in ICP during FESS and reinforces the hypothesis that the preferred anesthesia in this study might have protected from intracranial hypertension and related adverse effects.

With the direct infusion of saline solution over the dural sac, even when a pressure-controlled irrigation pump is used, an increase in ICP is expected^57^^–^ 59. However, there were nonsignificant variations in ICP waveform among patients that could be attributed to irrigation flow rate and the documented pressure in the pump. It is important to note that the irrigation system during FESS is considered open most of the time: i.e., the irrigation fluid enters the surgical field through an irrigation channel and exits through several ports, including the working cannula, the working channel, and the drainage channel, present in some endoscopes. Different FESS setups and endoscope brands may also interfere with the documented pressure in operating field, and with the irrigation flow. If the endoscope has an obstruction in the irrigation channel by surgical debris, the resistance will increase, as well as the documented pressure in the pump, but the irrigation flow will not correspond to the imputed value. Therefore, it is not possible to affirm that the irrigation flow or the imputed pressure does not affect the ICP since it is not possible to confirm that the parameters in the pump were faithfully affecting the epidural space. Additionally, ICP rise during FESS might happen rapidly and for short periods, for instance during occlusion of outflow channels^57^, and the noninvasive ICP monitoring method might not be suitable to identify those quick variations.

It has been reported that irrigation times exceeding 100 min are related to epileptic seizures and other adverse effects^9^^,^ 10, while irrigation rates above 150 mL/min are related to higher proportions of symptomatic patients during the surgical procedures and at the end of it^5^. Although in this study the mean total irrigation time was 100 min, there were only three symptomatic cases (patients 5, 9, and 31) in which intraoperative data did not show an increase in the P2/P1 ratio or TTP either. They have all related headaches after the procedure, without any other symptoms. These patients also showed a decrease in n-ICP parameters, meaning that symptoms might be related to other factors than intracranial hypertension or that momentary and harmless increases of ICP were not identified with the present method.

### Study limitations

Assigning information from a new method for ICP monitoring should raise many concerns. However, there is no other acceptable method to non-invasively check and test ICP throughout FESS.

In this study, we had a higher proportion of patients undergoing the interlaminar approach and under general anesthesia partially because general anesthesia is more comfortable for the patient and for the surgeon and did not increase adverse effects such as neuropraxia. In the authors’ personal experience, in the first years of spinal endoscopic practice, surgeries were all performed with local anesthesia, and it was quite common for patients to complain of headaches and neck pain, sometimes even preventing the surgery to be completed. Together with the literature that reports such complications with local and sevoflurane-based anesthesia, and the decrease of the n-ICP parameters when an increase was expected in this study, it is reasonable to hypothesize that general anesthesia and specific anesthetic drugs seem to protect from intracranial hypertension. Even though it is difficult to adopt the same anesthetic strategy as each patient have different demands, further studies comparing different anesthetic strategies might clarify this hypothesis. 

## Conclusion

The present study showed that n-ICP waveform parameters in individuals undergoing FESS significantly decreased after anesthetic induction and throughout the entire procedure when an increase in ICP was expected, showing a possible protective effect of anesthesia. The behavior of the studied ICP curve parameters was independent of the flow rate or pressure of the saline solution infused throughout surgery.

## Data Availability

All data sets were generated or analyzed in the current study.
